# Geometric Insights into the Multivariate Gaussian Distribution and Its Entropy and Mutual Information

**DOI:** 10.3390/e25081177

**Published:** 2023-08-07

**Authors:** Dah-Jing Jwo, Ta-Shun Cho, Amita Biswal

**Affiliations:** 1 Department of Communications, Navigation and Control Engineering, National Taiwan Ocean University, 2 Peining Rd., Keelung 202301, Taiwan; amitabiswal1988@gmail.com; 2 Department of Business Administration, Asia University, 500 Liufeng Road, Wufeng, Taichung 41354, Taiwan; cho2022@asia.edu.tw

**Keywords:** multivariate Gaussians, correlated random variables, visualization, entropy, relative entropy, mutual information

## Abstract

In this paper, we provide geometric insights with visualization into the multivariate Gaussian distribution and its entropy and mutual information. In order to develop the multivariate Gaussian distribution with entropy and mutual information, several significant methodologies are presented through the discussion, supported by illustrations, both technically and statistically. The paper examines broad measurements of structure for the Gaussian distributions, which show that they can be described in terms of the information theory between the given covariance matrix and correlated random variables (in terms of relative entropy). The content obtained allows readers to better perceive concepts, comprehend techniques, and properly execute software programs for future study on the topic’s science and implementations. It also helps readers grasp the themes’ fundamental concepts to study the application of multivariate sets of data in Gaussian distribution. The simulation results also convey the behavior of different elliptical interpretations based on the multivariate Gaussian distribution with entropy for real-world applications in our daily lives, including information coding, nonlinear signal detection, etc. Involving the relative entropy and mutual information as well as the potential correlated covariance analysis, a wide range of information is addressed, including basic application concerns as well as clinical diagnostics to detect the multi-disease effects.

## 1. Introduction

Understanding the ways knowledge concerning an external variable or the reciprocal information of its parts can assist in characterizing and inferring the underlying mechanics and function of the system. This goal has driven the development of several techniques for dissecting the elements of a set of variables’ combined entropy or for dissecting the contributions of a set of variables to the mutual information about the variable of interest. In actuality, this association and its modifications exist for any input signal and the widest range of Gaussian pathways, comprising discrete-time and continuous-time pathways in scalar or vector forms.

In a more general way, mutual information and mean-square error (MSE) are the fundamental concepts of information theory and estimation theory, respectively. In contrast to the minimum MSE (MMSE), which determines how precisely each input sample can be restored using the channel’s outcomes, the input-output mutual information is an estimation of whether the information can be consistently delivered over a channel given a specific input signal. An inactive functioning characterization for mutual information is provided by the substantial relevance of mutual information to estimation and filtering. Therefore, the significance of identity is not only obvious, but the link is also fascinating and merits an in-depth explanation [1,2,3]. Relations between the MMSE of the approximation of the output given the input and the localized actions of the mutual information at diminishing signal-to-noise ratio (SNR) are presented in [4]. The authors of [5] give an idea about the probabilistic ratios of geometric characteristics of signal detection in Gaussian noise. Furthermore, whether in a continuous-time [5,6,7] or discrete-time [8] context, the likelihood ratio is difficult in the relationship between observation and estimation.

Considering the specific instance of parametric computation (or Gaussian inputs), correlations relating to causal and non-causal estimation errors have been investigated in [9,10], involving the limit on the loss owing to the causality restriction. Knowing how data pertaining to an external parameter, or inversely related data within its parts, distributes across the parts of a multivariate system can assist in categorizing and determining the fundamental mechanics and functionality of the structure. The mechanism served as the impetus for the development of various techniques for decomposing the various elements of a set of parameters’ joint entropy [11,12,13,14,15,16,17,18,19] or for deconvoluting the additions of a set of elements to the mutual information about a target variable [13]. The mutual information techniques can be used to examine a variety of intricate systems, including those in the physical distinction domain, such as gene networks [17] or brain coding [20], as well as those in the social domain, such as selection agents [21] and community behavior [22]. It can also be used to analyze artificial agents [23]. Furthermore, some new proposals deviated more significantly from the original framework by incorporating novel principles such as the consideration of the presence of harmful elements associated with errors and the use of joint entropy subdivisions in place of mutual information [24,25].

In the multivariate scenario, the challenges of breaking down mutual information into redundancy and complementary sections have nevertheless been significantly increased. The maximum entropy framework allows for a more straightforward generalization of the efficiency measurements to the multivariate case [26,27]. The novel redundancy determines that were initially developed are only defined for the bivariate situation or allow negative components [28], whereas measurements of coordination are more readily extended to the multivariate case, especially when using the maximum entropy architecture [29,30]. By either utilizing the associations between lattices formed by various numbers of parameters or utilizing the multiple interactions between redundant lattices and information loss lattices, for which collaborative efforts are more actually defined, the study in [31,32] established two analogous techniques for constructing multivariate redundant metrics. Information theory variables have a benefit compared to more known test results measurements in that they may be employed when numerous ailments are being considered as well as when a test of diagnosis can produce several or continuous findings [33].

Although there are some valuable references detailing entropy-related topics, both discrete and continuous, they may not be easily accessible to some readers from the existing publications. Therefore, in this present study, we propose an extension of the bivariate Gaussian distribution technique to calculate multivariate redundant metrics inside the maximum entropy context. The importance of the maximum entropy approach in the multivariate scenario, where it offers constraints for the actual redundancy, unique information, and efficiency terms under logical presumptions shared by additional criteria, acts as the motivation for this particular focus [26,34]. The maximum entropy measurements, specifically, offer a lower limit for the actual cooperation and redundant terms and a higher limit for the actual specific information if it is presumed that a bivariate non-negative disintegration exists and that redundancy can be calculated from the bivariate distributions of the desired outcome with every source. Furthermore, if these bivariate distributions are consistent with possibly having little interaction under the previous hypotheses, then the maximum entropy decomposition returns not only boundaries but also the precise actual terms. Here, in the proposed framework, we also demonstrated that, under similar presumptions, the maximum entropy reduction also plays this dominant role in the multivariate situation [35]. This paper intends to convey the important issues and inspire new applications of information theory to a number of areas, such as information coding, nonlinear signal detection, and clinical diagnostic testing.

The remainder of this paper is organized as follows. A brief review of the geometry of the Gaussian distribution is reviewed in Section 2. The three consecutive sections deal with various important topics on information entropy with illustrative examples, with an emphasis on visualization of the information and discussion. In Section 3, continuous entropy/differential entropy are presented. In Section 4, the relative entropy (Kullback–Leibler divergence) is presented. Mutual information is presented in Section 5. Conclusions are given in Section 6.

## 2. Geometry of the Gaussian Distribution

In this section, the background relations of the Gaussian distribution from different parametric points of view will be discussed. The exploratory objective of the fundamental analysis is to identify “the framework” in multivariate datasets. Ordinary least-squares regression and principal component analysis (PCA), respectively, analyze the measurements for dependency (the predicted connection between particular components) and rigidity (the degree of prominence of the probability density function (pdf) around a low-dimensional axis) for bivariate Gaussian distributions. Mutual information, an established measure of dependency, is not an accurate indicator of rigidity since it is not invariant with an opposite rotation of the parameters. For bivariate Gaussian distributions, a suitable rotating invariant compactness measure is constructed and demonstrated to reduce the corresponding PCA measure.

### 2.1. Standard Parametric Representation of an Ellipse

For the uncorrelated data, which has zero covariance, the ellipse is not rotated and the axis is aligned. The radii of the ellipse in both directions are the variances. Geometrically, a not-rotated ellipse at point (0, 0) and radii a and b for the $x_{1}$- and $x_{2}$-direction is described by:

(1) $${\left(\frac{x_{1}}{a}\right)}^{2}+{\left(\frac{x_{2}}{b}\right)}^{2}=1.$$

The general probability density function for the multivariate Gaussian is given by the following:

(2) $$f_{X}(x|\mu ,\sum )=\frac{1}{{\left(\sqrt{2\pi }\right)}^{n}|\sum {|}^{1/2}}e^{\left\{-\frac{1}{2}{\left(X-\mu \right)}^{T}\sum^{-1}(X-\mu )\right\}},$$

where μ=E[X] and $\sum =\mathrm{Cov}(X)=E[(X-\mu ){\left(X-\mu \right)}^{T}]$ is a symmetric, positive semi-definite matrix. If ∑ is the identity matrix, then the Mahalanobis distance reduces to the standard Euclidean distance between X and μ.

For bivariate Gaussian distributions, the mean and covariance matrix are given by the following:

(3) $$\mu =\left[\begin{array}{c} \mu_{1}\\ \mu_{2} \end{array}\right];\;\sum =\left[\begin{array}{cc} \sigma_{1}^{2} & \sigma_{12}\\ \sigma_{12} & \sigma_{2}^{2} \end{array}\right]=\left[\begin{array}{cc} \sigma_{1}^{2} & \rho \sigma_{1}\sigma_{2}\\ \rho \sigma_{1}\sigma_{2} & \sigma_{2}^{2} \end{array}\right],$$

where the linear correlation coefficient |ρ|≤1.

Variance measures the variation of a single random variable, whereas covariance is a measure of the two random variables varying together. With the covariance, we can calculate the entries of the covariance matrix, which is a square matrix. In addition, the covariance matrix is symmetric. The diagonal entries of the covariance matrix are the variances; however, the other entries are the covariances. Due to this reason, the covariance matrix is often called the variance-covariance matrix.

### 2.2. The Confidence Ellipse

A typical way to visualize two-dimensional Gaussian-distributed data is by plotting a confidence ellipse. The distance $d_{M}={\left(X-\mu \right)}^{T}\sum^{-1}(X-\mu )$ is a constant value referred to as the Mahalanobis distance, which is a random variable distributed by the chi-squared distribution, denoted as $\chi_{k}^{2}$.

(4) $$P[{\left(X-\mu \right)}^{T}\sum^{-1}(X-\mu )\leq \chi_{k}^{2}(\alpha )]=1-\alpha ,$$

where k is the number of degrees of freedom and α is the given probability related to the confidence ellipse. For example, if α=0.95, 95% confidence ellipse is defined. Extension from Equation (1): the radius in each direction is the standard deviation $\sigma_{1}$ and $\sigma_{2}$ parametrized by a scale factor *s*, known as the Mahalanobis radius of the ellipsoid:

(5) $${\left(\frac{x_{1}}{\sigma_{1}}\right)}^{2}+{\left(\frac{x_{2}}{\sigma_{2}}\right)}^{2}=s.$$

The goal is to determine the scale *s* such that confidence *p* is met. Since the data are multivariate Gaussian-distributed, the left-hand side of the equation is the sum of squares of Gaussian-distributed samples, which follows a *χ*^2^ distribution. A *χ*^2^ distribution is defined by the degrees of freedom, and since we have two dimensions, the number of degrees of freedom is two. Now, we have calculated the probability with the sum, and therefore *s* has a certain value under a *χ*^2^ distribution.

**Figure 1:**
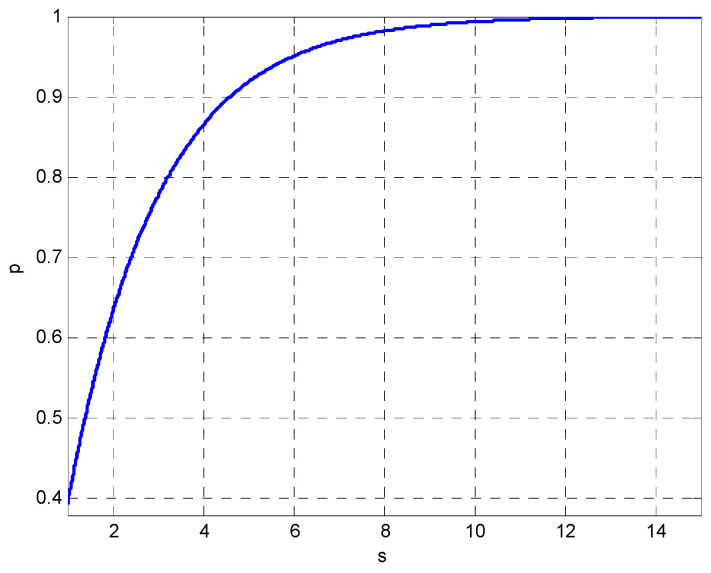
Relationship between the confidence interval and the scale factor *s*.

This ellipse with a probability contour defines the region of a minimum area (or volume in the multivariate case) containing a given probability under the Gaussian assumption. The equation can be solved using a *χ*^2^ table or simply using the relationship s=−2ln(1−p). The confidence interval can be evaluated through the following:

(6) $$p=1-exp\left(-0.5s\right).$$

for s=1 we have $p=1-exp\left(-0.5\right)\approx 0.3935$. Furthermore, typical values include s=2.279, s=4.605, s=5.991, and s=9.210 for p=0.68, p=0.9, p=0.95, and p=0.99, respectively. The ellipse can then be drawn with radii $\sigma_{1}\sqrt{s}$ and $\sigma_{2}\sqrt{s}$. Figure 1 shows the relationship between the confidence interval and the scale factor *s*.

The Mahalanobis distance accounts for the variance of each variable and the covariance between variables.

(7) $$\begin{array}{l} {\left(X-\mu \right)}^{T}\sum^{-1}(X-\mu )\\ =\left[\begin{array}{cc} x_{1}-\mu_{1} & x_{2}-\mu_{2} \end{array}\right]{\left[\begin{array}{cc} \sigma_{1}^{2} & \rho \sigma_{1}\sigma_{2}\\ \rho \sigma_{1}\sigma_{2} & \sigma_{2}^{2} \end{array}\right]}^{-1}\left[\begin{array}{c} x_{1}-\mu_{1}\\ x_{2}-\mu_{2} \end{array}\right]\\ =\left[\begin{array}{cc} x_{1}-\mu_{1} & x_{2}-\mu_{2} \end{array}\right]\frac{\left[\begin{array}{cc} \sigma_{2}^{2} & -\rho \sigma_{1}\sigma_{2}\\ \rho \sigma_{1}\sigma_{2} & \sigma_{1}^{2} \end{array}\right]}{\sigma_{1}^{2}\sigma_{2}^{2}(1-\rho^{2})}\left[\begin{array}{c} x_{1}-\mu_{1}\\ x_{2}-\mu_{2} \end{array}\right]\\ =\frac{1}{1-\rho^{2}}\left(\frac{{\left(x_{1}-\mu_{1}\right)}^{2}}{\sigma_{1}^{2}}-\frac{2\rho (x_{1}-\mu_{1})(x_{2}-\mu_{2})}{\sigma_{1}\sigma_{2}}+\frac{{\left(x_{2}-\mu_{2}\right)}^{2}}{\sigma_{2}^{2}}\right) \end{array}.$$

Geometrically, it does this by transforming the data into standardized, uncorrelated data and computing the ordinary Euclidean distance for the transformed data. In this way, the Mahalanobis distance is like a univariate z-score: it provides a way to measure distances that takes into account the scale of the data.

In the general case, covariances $\sigma_{12}$ and $\sigma_{21}$ are not zero, and therefore the ellipse-coordinate system is not axis-aligned. In such a case, instead of using the variance as a spread indicator, we use the eigenvalues of the covariance matrix. The eigenvalues represent the spread in the direction of the eigenvectors, which are the variances under a rotated coordinate system. By definition, a covariance matrix is positive and definite; therefore, all eigenvalues are positive and can be seen as a linear transformation of the data. The actual radii of the ellipse are $\sqrt{\lambda_{1}}$ and $\sqrt{\lambda_{2}}$ for the two eigenvalues $\lambda_{1}$ and $\lambda_{2}$ of the scaled covariance matrix s⋅∑.

Based on Equations (2) and (7), the bivariate Gaussian distributions can be represented as follows:

(8) $$f(x_{1},x_{2})=\frac{1}{2\pi \sigma_{1}\sigma_{2}\sqrt{1-\rho^{2}}}e^{-\frac{1}{2}\left(\frac{1}{1-\rho^{2}}\right)\left\{\frac{{\left(x_{1}-\mu_{1}\right)}^{2}}{\sigma_{1}^{2}}-2\rho \frac{\left(x_{1}-\mu_{1})(x_{2}-\mu_{2}\right)}{\sigma_{1}\sigma_{2}}+\frac{{\left(x_{2}-\mu_{2}\right)}^{2}}{\sigma_{2}^{2}}\right\}},$$

and the level surface of $f(x_{1},x_{2})$ are concentric ellipses:

(9) $$\frac{{\left(x_{1}-\mu_{1}\right)}^{2}}{\sigma_{1}^{2}}-2\rho \frac{\left(x_{1}-\mu_{1})(x_{2}-\mu_{2}\right)}{\sigma_{1}\sigma_{2}}+\frac{{\left(x_{2}-\mu_{2}\right)}^{2}}{\sigma_{2}^{2}}=c,$$

where c is the Mahalanobis distance possessing the following properties:

▪ It accounts for the fact that the variances in each direction are different;
▪ It accounts for the covariance between variables;
▪ It reduces to the familiar Euclidean distance for uncorrelated variables with unit variance.

The length of the ellipse axes is a function of the given probability of the chi-squared distribution with 2 degrees of freedom $\chi_{2}^{2}(\alpha )$, the eigenvalues $\lambda ={\left[\begin{array}{cc} \lambda_{1} & \lambda_{2} \end{array}\right]}^{T}$ and the linear correlation coefficient ρ. If α=0.95, 95% confidence ellipse is defined by:

(10) $$\left[\begin{array}{cc} x_{1}-\mu_{1} & x_{2}-\mu_{2} \end{array}\right]\sum^{-1}\left[\begin{array}{c} x_{1}-\mu_{1}\\ x_{2}-\mu_{2} \end{array}\right]\leq \chi_{2}^{2}(0.05)$$

where

(11) $$\sum^{-1}=\frac{1}{\sigma_{1}^{2}\sigma_{2}^{2}(1-\rho^{2})}\left[\begin{array}{cc} \sigma_{2}^{2} & -\rho \sigma_{1}\sigma_{2}\\ \rho \sigma_{1}\sigma_{2} & \sigma_{1}^{2} \end{array}\right],$$

as ∑ denotes a symmetric matrix, the eigenvectors of ∑ is linearly independent (or orthogonal).

### 2.3. Similarity Transform

The simplest similarity transformation method for eigenvalue computation is the Jacobi method, which deals with the standard eigenproblems. In the multivariate Gaussian distribution, the covariance matrix ∑ can be expressed in terms of eigenvectors:

(12) $$\sum =U\Lambda U^{-1}=U\Lambda U^{T}=\left[\begin{array}{cc} u_{1} & u_{2} \end{array}\right]\left[\begin{array}{cc} \lambda_{1} & 0\\ 0 & \lambda_{2} \end{array}\right]\left[\begin{array}{c} u_{1}^{T}\\ u_{2}^{T} \end{array}\right],$$

where $U=\left[\begin{array}{cc} u_{1} & u_{2} \end{array}\right]$ are the eigenvectors of ∑ and Λ is the diagonal matrix of the eigenvalues $\lambda ={\left[\begin{array}{cc} \lambda_{1} & \lambda_{2} \end{array}\right]}^{T}$

$$\Lambda =\left[\begin{array}{cc} \lambda_{1} & 0\\ 0 & \lambda_{2} \end{array}\right],$$

replacing ∑ by $\sum^{-1}=U\Lambda^{-1}U^{-1}$, the square of the difference can be written as:

(13) $$\left[\begin{array}{cc} x_{1}-\mu_{1} & x_{2}-\mu_{2} \end{array}\right]U\Lambda^{-1}U^{-1}\left[\begin{array}{c} x_{1}-\mu_{1}\\ x_{2}-\mu_{2} \end{array}\right]\leq \chi_{2}^{2}(0.05),$$

as $U^{T}=U^{-1}$. Denoting

(14) $$\left[\begin{array}{c} y_{1}\\ y_{2} \end{array}\right]=U^{-1}\left[\begin{array}{c} x_{1}-\mu_{1}\\ x_{2}-\mu_{2} \end{array}\right],$$

the square of the difference can then be expressed as:

(15) $$\left[\begin{array}{cc} y_{1} & y_{2} \end{array}\right]\left[\begin{array}{cc} \lambda_{1} & 0\\ 0 & \lambda_{2} \end{array}\right]\left[\begin{array}{c} y_{1}\\ y_{2} \end{array}\right]\leq \chi_{2}^{2}(0.05).$$

If the above equation is further evaluated, the resulting equation is the equation of an ellipse aligned with the axis $y_{1}$ and $y_{2}$ in the new coordinate system:

(16) $$\frac{y_{1}^{2}}{\chi_{2}^{2}(0.05)\lambda_{1}}+\frac{y_{2}^{2}}{\chi_{2}^{2}(0.05)\lambda_{2}}\leq 1,$$

the axes of the ellipse are defined by $y_{1}$ axis with a length $2\sqrt{\lambda_{1}\chi_{2}^{2}(0.05)}$ and $y_{2}$ axis with a length $2\sqrt{\lambda_{2}\chi_{2}^{2}(0.05)}$.

When ρ=0, the eigenvectors are equal to $\lambda_{1}=\sigma_{1}$ and $\lambda_{2}=\sigma_{2}$. Additionally, U matrix whose elements are the eigenvectors, of ∑ becomes an identity matrix. The final equation of an ellipse is then defined by:

(17) $$\frac{{\left(x_{1}-\mu_{1}\right)}^{2}}{\chi_{2}^{2}(0.05)\lambda_{1}}+\frac{{\left(x_{2}-\mu_{2}\right)}^{2}}{\chi_{2}^{2}(0.05)\lambda_{2}}\leq 1.$$

It is clear from the equation given above that the axes of the ellipse are parallel to the coordinate axes. The lengths of the axes of the ellipse are then defined as $2\sqrt{\sigma_{11}\chi_{2}^{2}(0.05)}$ and $2\sqrt{\sigma_{22}\chi_{2}^{2}(0.05)}$.

The covariance matrix can be presented by its eigenvectors and eigenvalues: ∑U=UΛ, where U is the matrix whose columns are the eigenvectors of ∑ and Λ is the diagonal matrix with diagonal elements given by the eigenvalues of ∑. Transformation is performed based on the three steps involving scaling, rotation, and translation:

1. Scaling

The covariance matrix can be written as $\sum =U\Lambda U^{-1}=USSU^{-1}$, where S is a diagonal scaling matrix $S=\Lambda^{1/2}=S^{T}$;

2. Rotation

U is generalized from the normalized eigenvectors of the covariance matrix ∑.

(18) $$U=\left[\begin{array}{cc} \cos(\theta )\; & -\sin(\theta )\\ \sin(\theta ) & \cos(\theta ) \end{array}\right],$$

it can be noted that U is an orthogonal matrix $U^{-1}=U^{T}$ and |U|=1. Here, we have calculated the matrix with rotation and scaling T=US and $T^{T}={\left(US\right)}^{T}=S^{T}U^{T}=SU^{-1}$. Thus, the covariance matrix can be written as $\sum =TT^{T}$ and $U^{T}\sum U=\Lambda$ with diagonal eigenvalues $\lambda_{i}$. Since T=US, we have $Y=TX=USX=U\Lambda^{1/2}X$.

(19) $$\left[\begin{array}{c} x_{1}(t)\\ x_{2}(t) \end{array}\right]=\left[\begin{array}{cc} u_{1x} & u_{2x}\\ u_{1y} & u_{2y} \end{array}\right]\left[\begin{array}{c} \sqrt{\lambda_{1}}\cos(t)\\ \sqrt{\lambda_{2}}\sin(t) \end{array}\right]=\left[\begin{array}{cc} \cos(\theta )\; & -\sin(\theta )\\ \sin(\theta ) & \cos(\theta ) \end{array}\right]\left[\begin{array}{c} \sqrt{\lambda_{1}}\cos(t)\\ \sqrt{\lambda_{2}}\sin(t) \end{array}\right]$$

The similarity transform is applied to obtain the relationship between $X^{T}\sum^{-1}X=Y^{T}U^{T}\sum^{-1}UY=Y^{T}\Lambda^{-1}Y$, and the pdf of Y vector, which can be found by considering the below expression:

(20) $$f_{Y}(y)=\prod_{i=1}^{n}\frac{1}{\sqrt{2\pi }\sqrt{\lambda_{i}}}e^{-\frac{1}{2}\frac{y_{i}^{2}}{\lambda_{i}}},$$

the ellipse in the transformed frame can be represented as:

(21) $$\frac{y_{1}^{2}}{\lambda_{1}}+\frac{y_{2}^{2}}{\lambda_{2}}=c,$$

where the eigenvectors are equal to $\lambda_{1}=\sigma_{1}^{2}$ and $\lambda_{2}=\sigma_{2}^{2}$;

3. Translation

(22) $$x_{1}(t)=\sqrt{\lambda_{1}}\cos(\theta )\cos(t)-\sqrt{\lambda_{2}}\sin(\theta )\sin(t)+\mu_{1},$$

(23) $$x_{2}(t)=\sqrt{\lambda_{1}}\sin(\theta )\cos(t)+\sqrt{\lambda_{2}}\cos(\theta )\sin(t)+\mu_{2},$$

the eigenvalues $\lambda ={\left[\begin{array}{cc} \lambda_{1} & \lambda_{2} \end{array}\right]}^{T}$ can be calculated from:

$$\lambda_{1}=\frac{1}{2}\left[\sigma_{1}^{2}+\sigma_{2}^{2}+\sqrt{{\left(\sigma_{1}^{2}-\sigma_{2}^{2}\right)}^{2}+4\rho^{2}\sigma_{1}^{2}\sigma_{2}^{2}}\right];\;\lambda_{2}=\frac{1}{2}\left[\sigma_{1}^{2}+\sigma_{2}^{2}-\sqrt{{\left(\sigma_{1}^{2}-\sigma_{2}^{2}\right)}^{2}+4\rho^{2}\sigma_{1}^{2}\sigma_{2}^{2}}\right],$$

and thus

(24) $$|\sum |=\lambda_{1}\cdot \lambda_{2}=\sigma_{1}^{2}\sigma_{2}^{2}(1-\rho^{2}).$$

From another point of view, the covariance matrix can be calculated as:

(25) $$\begin{array}{c} \sum =U\Lambda U^{T}=\left[\begin{array}{cc} \cos(\theta )\; & -\sin(\theta )\\ \sin(\theta ) & \cos(\theta ) \end{array}\right]\left[\begin{array}{cc} \lambda_{1} & 0\\ 0 & \lambda_{2} \end{array}\right]\left[\begin{array}{cc} \cos(\theta )\; & \sin(\theta )\\ -\sin(\theta ) & \cos(\theta ) \end{array}\right],\\ =\left[\begin{array}{cc} \lambda_{1}\cos(\theta )\; & -\lambda_{2}\sin(\theta )\\ \lambda_{1}\sin(\theta ) & \lambda_{2}\cos(\theta ) \end{array}\right]\left[\begin{array}{cc} \cos(\theta )\; & \sin(\theta )\\ -\sin(\theta ) & \cos(\theta ) \end{array}\right],\\ =\left[\begin{array}{cc} \lambda_{1}\cos^{2}(\theta )+\lambda_{2}\sin^{2}(\theta )\; & \left(\lambda_{1}-\lambda_{2})(\sin(\theta )-\cos(\theta )\right)\\ syms & \lambda_{1}\sin^{2}(\theta )+\lambda_{2}\cos^{2}(\theta ) \end{array}\right]. \end{array}$$

Calculation for the determinant of the above covariance matrix gives the same result, and the inverse is:

(26) $$\begin{array}{c} \sum^{-1}=\frac{1}{\lambda_{1}\cdot \lambda_{2}}\left[\begin{array}{cc} \lambda_{1}\sin^{2}(\theta )+\lambda_{2}\cos^{2}(\theta )\; & \left(\lambda_{2}-\lambda_{1})(\sin(\theta )-\cos(\theta )\right)\\ syms & \lambda_{1}\cos^{2}(\theta )+\lambda_{2}\sin^{2}(\theta ) \end{array}\right],\\ =\left[\begin{array}{cc} \frac{\sin^{2}(\theta )}{\lambda_{2}}+\frac{\cos^{2}(\theta )}{\lambda_{1}} & \sin(\theta )\cos(\theta )\left(\frac{1}{\lambda_{1}}-\frac{1}{\lambda_{2}}\right)\\ syms & \frac{\sin^{2}(\theta )}{\lambda_{1}}+\frac{\sin^{2}(\theta )}{\lambda_{2}} \end{array}\right]. \end{array}$$

### 2.4. Simulation with a Given Variance-Covariance Matrix

With the given data X~N(μ,∑), an ellipse represents the confidence *p,* which can be plotted by calculating the radii, its center, and the rotation. Here, θ (by which U can be obtained) and S for generating the covariance matrix ∑, from which ρ can be derived. The inclination angle is calculated by:

(27) $$\theta =\left\{\begin{array}{cc} 0 & if\;\sigma_{12}=0\;and\;\sigma_{1}^{2}\geq \sigma_{2}^{2}\\ \pi /2 & if\;\sigma_{12}=0\;and\;\sigma_{1}^{2}<\sigma_{2}^{2}\\ \tan^{-1}(\lambda_{1}-\sigma_{1}^{2},\sigma_{12}) & else \end{array}\right.,$$

which can be used in calculations with the values of U

(28) $$U=\left[\begin{array}{cc} \cos(\theta )\; & -\sin(\theta )\\ \sin(\theta ) & \cos(\theta ) \end{array}\right],$$

and the covariance can be evaluated by: $\sum =U\Lambda U^{T}=USSU^{T}$ if S is specified. On the other hand, with the correlation coefficient ρ and variances for generating the covariance matrix ∑, θ can be obtained.

To generate the sampling points that meet the specified correlation, the following procedure can be followed. Given two random variables $X_{1}$ and $X_{2}$, their linear combination is $Y=\alpha X_{1}+\beta X_{2}$. For the generation of correlated random variables, if we have two Gaussian, uncorrelated random variables $X_{1}$, $X_{2}$ then we can create two correlated random variables using the formula:

(29) $$Y=\rho X_{1}+\sqrt{1-\rho^{2}}X_{2},$$

and then Y will have a correlation ρ with $X_{1}$:

$$\rho =\sigma_{12}/(\sigma_{1}\sigma_{2}).$$

Based on the relationship: X=AZ+μ, Z~N(0,1), the following equation can be employed to generate the sampling points for the scatter plots using the MATLAB software:

(30) X=A*randn(2,K)+μ*ones(1,K),

where the Cholesky decomposition of ∑ has a lower triangular matrix for A, $\sum =AA^{T}$ and μ is the vector of mean values.

**Figure 2:**
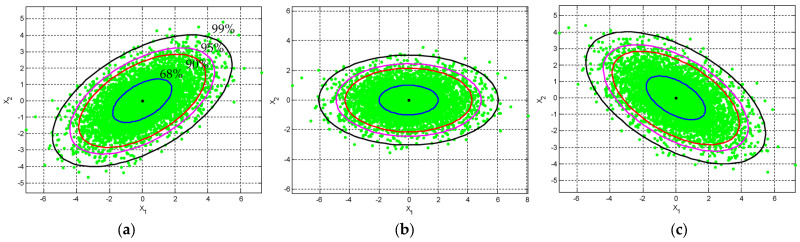
The position of the ellipse with various correlation coefficients given by the angle of inclination is specified θ to obtain ρ, $\rho =\sigma_{12}/(\sigma_{1}\sigma_{2})$: (**a**) $\theta =30^{\circ }$, ρ≈0.55; (**b**) $\theta =0^{\circ }$,ρ=0; and (**c**) $\theta =150^{\circ }$, ρ≈−0.55, respectively.

**Figure 3:**
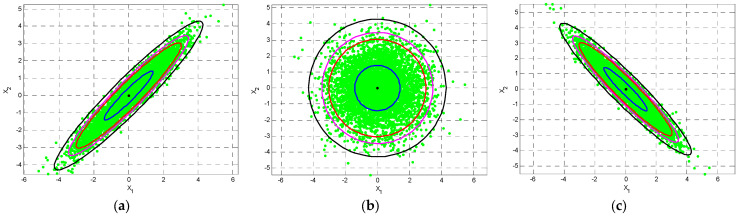
The position of the ellipse for various values of the correlation constant with the angle of inclination ρ is specified to obtain θ: (**a**) ρ=0.95, $\theta =45^{\circ }$; (**b**) ρ=0, $\theta =0^{\circ }$; and (**c**) ρ=−0.95, $\theta =135^{\circ }$, respectively.

When ρ=0, the axes of the ellipse are parallel to the original coordinate system, and when ρ≠0, the axes of the ellipse are aligned with the rotated axes in the transformed coordinate system. Figure 2 and Figure 3 show the ellipses for various levels of confidence. The plots provide the idea of confidence (error) ellipses with different confidence levels (i.e., 68%, s=2.279; 90%, s=4.605; 95%, s=5.991; and 99%, s=9.210) from inner to outer ellipses, respectively, by considering the cases where the random variables are: (1) positively correlated ρ>0, (2) negatively correlated ρ<0, and (3) independent ρ=0. More specifically, in Figure 2, the position of the ellipse with various correlation coefficients given by the angle of inclination is specified θ to obtain ρ, $\rho =\sigma_{12}/(\sigma_{1}\sigma_{2})$: (a) $\theta =30^{\circ }$, ρ≈0.55; (b) $\theta =0^{\circ }$, ρ=0; and (c) $\theta =150^{\circ }$, ρ≈−0.55, respectively. On the other hand, in Figure 3, the position of the ellipse with various values of the correlation constant given the angle of inclination is specified ρ to obtain θ: (a) $\rho =0.95^{\circ }$, $\theta =45^{\circ }$; (b) ρ=0, $\theta =0^{\circ }$; and (c) $\rho =-0.95^{\circ }$, $\theta =135^{\circ }$, respectively. The rotation angle is measured $0\leq \theta \leq 180^{\circ }$ with respect to the positive axis. When ρ>0, the angle is in the first quadrant and when ρ<0, the angle is in the second quadrant.

**Figure 4:**
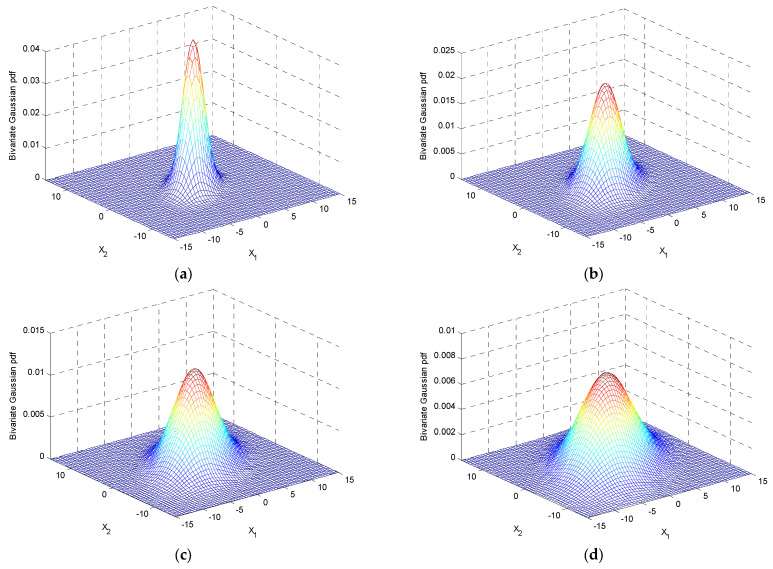
The contours and the scatter plots of ellipses for equal variances $\sigma_{1}=\sigma_{2}=\sigma$ with a fixed ρ=0.5: (**a**) σ=2 (**b**) σ=3 (**c**) σ=4 (**d**) σ=5.

In the following, two case studies involve more illustrations:

(1) Equal variances for two random variables with nonzero ρ:

Case 1: fixed correlation coefficient. As an example, when ρ=0.5, and the variances $\sigma_{1}=\sigma_{2}=\sigma$ range from 2~5, as shown in Figure 4. As can be seen, the contours and the scatter plots are ellipses instead of circles.

$$\sum =\left[\begin{array}{cc} \sigma_{1}^{2} & \sigma_{12}\\ \sigma_{21} & \sigma_{2}^{2} \end{array}\right]=\left[\begin{array}{cc} \sigma_{1}^{2} & \rho \sigma_{1}\sigma_{2}\\ \rho \sigma_{1}\sigma_{2} & \sigma_{2}^{2} \end{array}\right]=\left[\begin{array}{cc} 4^{2} & 0.5(4)(2)\\ 0.5(4)(2) & 2^{2} \end{array}\right]=\left[\begin{array}{cc} 4^{2} & 4\\ 4 & 2^{2} \end{array}\right]$$

**Figure 5:**
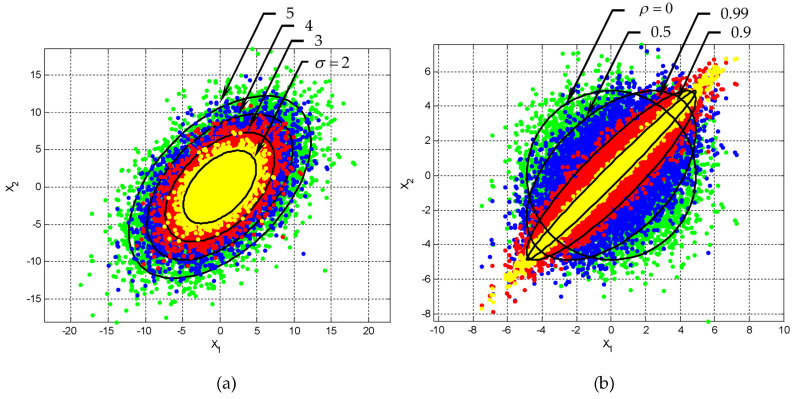
Ellipses for (**a**) ρ=0.5 with varying variances $\sigma_{1}=\sigma_{2}=\sigma =2\sim 5$; and (**b**) equal variances $\sigma_{1}=\sigma_{2}=2$ with varying ρ=0;0.5;0.9;0.99.

Subplot (a) in Figure 5 shows the ellipses for ρ=0.5 with varying variances. Here and in subsequent illustrations, 95% confidence levels are shown;

Case 2: increasing the correlation coefficient ρ from zero correlation. With fixed variance $\sigma_{1}=\sigma_{2}=\sigma$, the contour will initially be a circle when ρ=0 and then an ellipse as ρ increases when ρ≠0. Subplot (b) in Figure 5 provides the contours with scatter plots for ρ=0,0.5,0.9,0.99, respectively, when $\sigma_{1}=\sigma_{2}=2$. The eccentricity of the ellipses increases with the increase of ρ.

(2) Unequal variances for two random variables, $\sigma_{1}\neq \sigma_{2}$ with fixed correlation coefficient ρ=0.5.

**Figure 6:**
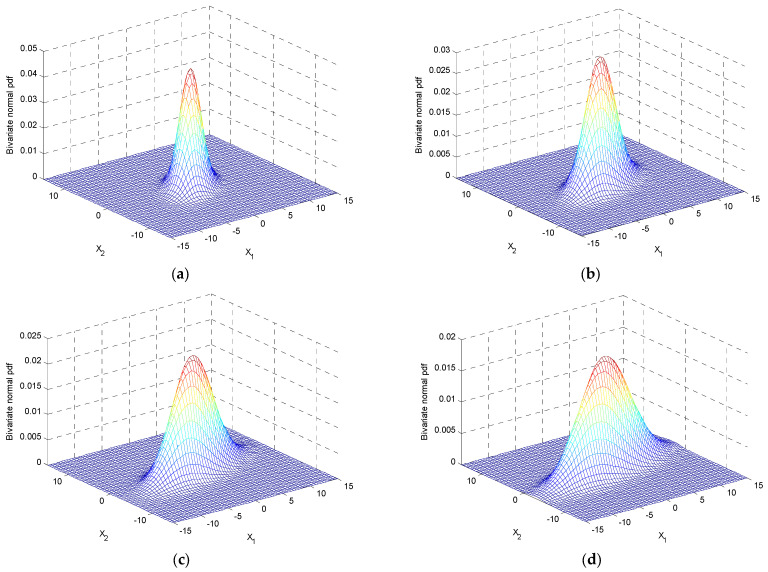
The variations of surface plots in three-dimensional with the increase in $\sigma_{1}/\sigma_{2}$ for a fixed ρ=0.5 where $\sigma_{2}=2$: (**a**) $\sigma_{1}=2$; (**b**) $\sigma_{1}=3$; (**c**) $\sigma_{1}=4$; and (**d**) $\sigma_{1}=5$.

**Figure 7:**
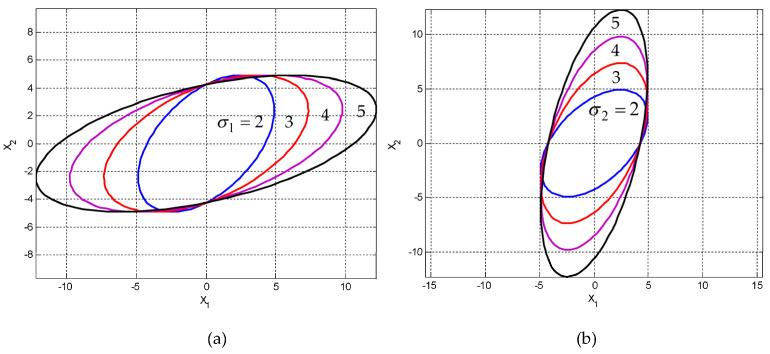
Ellipses for a fixed correlation coefficient when $\sigma_{1}\neq \sigma_{2}$ for a fixed ρ=0.5: (**a**) $\sigma_{1}>\sigma_{2}$, $\sigma_{1}/\sigma_{2}$ increases where $\sigma_{1}=2\sim 5$ and $\sigma_{2}=2$; and (**b**) $\sigma_{2}>\sigma_{1}$, $\sigma_{2}/\sigma_{1}$ increases where $\sigma_{2}=2\sim 5$ and $\sigma_{1}=2$.

Case 1: $\sigma_{1}>\sigma_{2}$. The variations of three-dimensional surfaces and ellipses are presented in Figure 6 and Figure 7a with the increase of $\sigma_{1}/\sigma_{2}$, where $\sigma_{1}=2\sim 5$ and $\sigma_{2}=2$.

**Figure 8:**
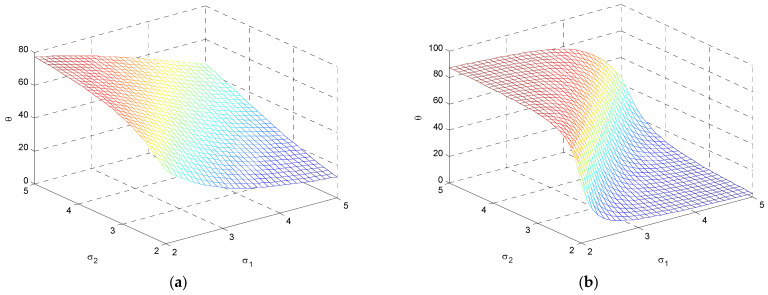
The variation for inclination angle with a function of $\sigma_{1}$ and $\sigma_{2}$, for (**a**) ρ=0.5; and (**b**) ρ=0.

Case 2: $\sigma_{2}>\sigma_{1}$. The variation of the ellipses is presented in Figure 7b with the increase of $\sigma_{2}/\sigma_{1}$, where $\sigma_{2}=2\sim 5$ and $\sigma_{1}=2$. Figure 8 shows the variation of inclination angle as a function of $\sigma_{1}$ and $\sigma_{2}$, for ρ=0 and ρ=0.5 for providing further insights on the variation of inclination angle θ with respect to $\sigma_{1}$ and $\sigma_{2}$.

**Figure 9:**
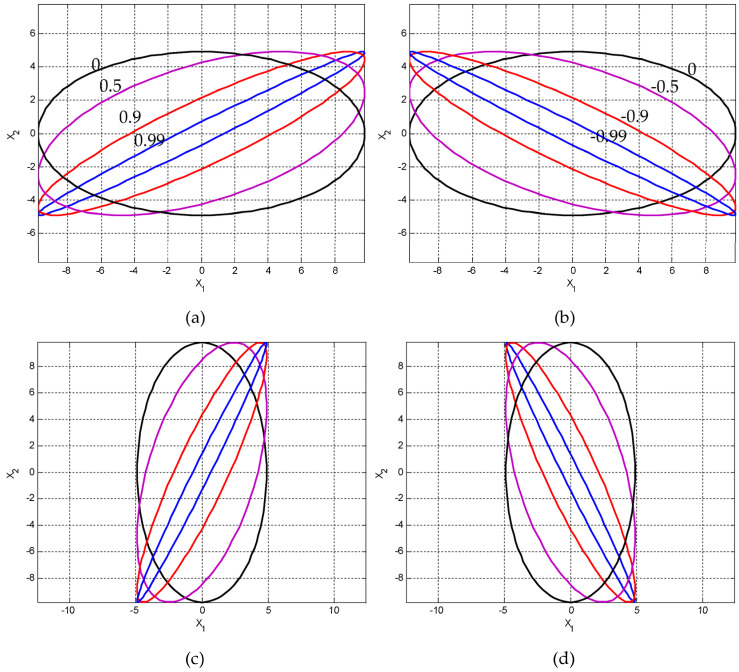
($\sigma_{1}=4$, $\sigma_{2}=2$) with (**a**) ρ=0,0.5,0.9,0.99; (**b**) ρ=0,−0.5,−0.9,−0.99 as compared to $\sigma_{2}>\sigma_{1}$ ($\sigma_{1}=2$, $\sigma_{2}=4$) with (**c**) ρ=0,0.5,0.9,0.99; and (**d**) ρ=0,−0.5,−0.9,−0.99.

**Figure 10:**
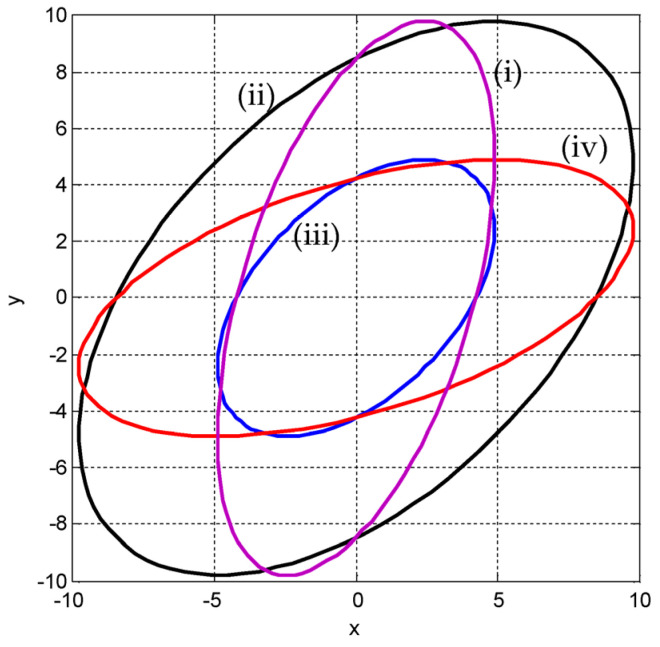
Comparison of the ellipses for various (i) $\sigma_{1}=2$, $\sigma_{2}=4$; (ii) $\sigma_{1}=4$, $\sigma_{2}=2$; (iii) $\sigma_{1}=\sigma_{2}=2$; and (iv) $\sigma_{1}=\sigma_{2}=4$, while ρ=0.5.

(3) Variation of the ellipses for the various positive and negative correlations. For a given variance, when ρ is specified, the eigenvalues and the inclination angle are obtained accordingly. Figure 9 presents results for the cases of $\sigma_{1}>\sigma_{2}$ ($\sigma_{1}=4$, $\sigma_{2}=2$ in this example) and $\sigma_{2}>\sigma_{1}$ ($\sigma_{1}=2$, $\sigma_{2}=4$ in this example) with various correlation coefficients (namely, positive, zero, and negative), including ρ=0,0.5,0.9,0.99 and ρ=0,−0.5,−0.9,−0.99. In the figure, $\sigma_{1}=4$, $\sigma_{2}=2$ are applied for the top plots, while $\sigma_{1}=2$,$\sigma_{2}=4$ are applied for the bottom plots. On the other hand, ρ=0,0.5,0.9,0.99 are applied for the left plots, while ρ=0,−0.5,−0.9,−0.99 are applied for the right plots. Furthermore, Figure 10 provides a comparison of the ellipses for various $\sigma_{1}$ and $\sigma_{2}$ for the following cases: (i) $\sigma_{1}=2$, $\sigma_{2}=4$; (ii) $\sigma_{1}=4$, $\sigma_{2}=2$; (iii) $\sigma_{1}=\sigma_{2}=2$; and (iv) $\sigma_{1}=\sigma_{2}=4$, while ρ=0.5.

## 3. Continuous Entropy/Differential Entropy

Differential entropy (also referred to as continuous entropy) is a concept in information theory that began as an attempt by Claude Shannon to extend the idea of (Shannon) entropy, a measure of the average surprise of a random variable, to continuous probability distributions. Unfortunately, Shannon did not derive this formula and rather just assumed it was the correct continuous analog of discrete entropy, but it is not [1]. The actual continuous version of discrete entropy is the limiting density of discrete points (LDDP). Differential entropy (described here) is commonly encountered in the literature, but it is a limiting case of the LDDP and one that loses its fundamental association with discrete entropy.

In the following discussion, differential entropy and relative entropy are measured in bits, which are used in the definition. Instead, if ln is used, it is then measured in nats, and the only difference in the expression is the $\log_{2}e$ factor.

### 3.1. Entropy of a Univariate Gaussian Distribution

If we have a continuous random variable X with a probability density function (pdf) $f_{X}(x)$, the differential entropy of X in bits is expressed as:

(31) $$h(X)=-E[\log_{2}f_{X}(x)]=-\int f_{X}(x)\log_{2}f_{X}(x)dx,$$

let X be a Gaussian random variable $X\sim N(\mu ,\sigma^{2})$

$$f_{X}(x)=\frac{1}{\sqrt{2\pi }\sigma }e^{-\frac{1}{2}{\left(\frac{x-\mu }{\sigma }\right)}^{2}}.$$

The differential entropy for this univariate Guassian distribution can be evaluated as follows:

(32) $$\begin{array}{l} h(X)\\ =-E[\log_{2}f_{X}(x)]\\ =-\int f_{X}(x)\log_{2}f_{X}(x)dx\\ =-\int f_{X}(x)\log_{2}\left(\frac{1}{\sqrt{2\pi }\sigma }e^{-\frac{{\left(x-\mu \right)}^{2}}{2\sigma^{2}}}\right)dx\\ =\frac{1}{2}\log_{2}(2\pi e\sigma^{2}) \end{array}.$$

**Figure 11:**
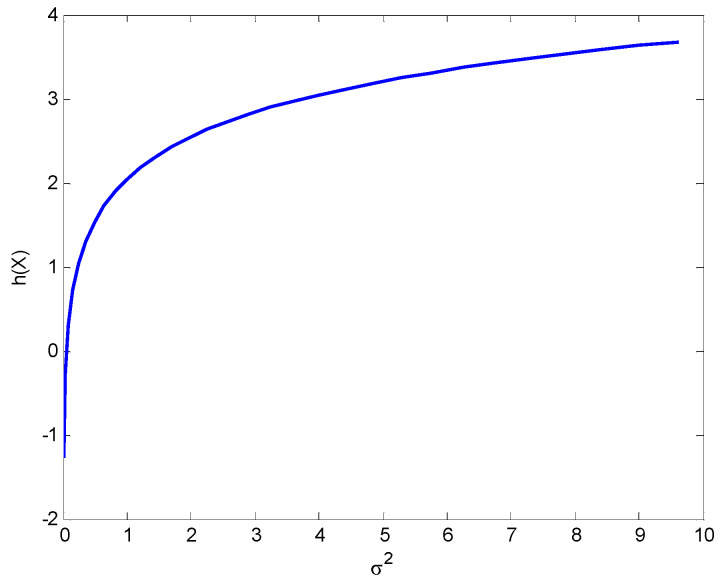
The differential entropy as a function of $\sigma^{2}$ for a univariate Gaussian variable.

Figure 11 shows the differential entropy as a function $\sigma^{2}$ for the univariate Gaussian variable, which is concave downward and grows first very fast and then much more slowly at high values of $\sigma^{2}$.

### 3.2. Entropy of a Multivariate Gaussian Distribution

Let X follow a *multivariate* Gaussian distribution X~N(μ,∑), as given by Equation (2), then the differential entropy of X in nats is:

(33) $$h(X)=-E[\log_{2}f_{X}(x)]=-\int f_{X}(x)\log_{2}f_{X}(x)dx,$$

and the differential entropy is given by Appendix B:

(34) $$h(X)=\frac{1}{2}\log_{2}({\left(2\pi e\right)}^{n}|\sum |).$$

The above calculation involves the evaluation of expectations of the Mahalanobis distance as (Appendix C):

(35) $$E[{\left(x-\mu \right)}^{T}\sum^{-1}(x-\mu )]=n.$$

For a fixed variance, the normal distribution is the pdf that maximizes entropy. Let $X={\left[\begin{array}{cc} X_{1} & X_{2} \end{array}\right]}^{T}$ be a 2D Gaussian vector, and the entropy of X can be calculated to be:

(36) $$h(X)=h(X_{1},X_{2})=\frac{1}{2}\log_{2}\left({\left(2\pi e\right)}^{2}|\sum |\right)=\log_{2}(2\pi e\sigma_{1}\sigma_{2}\sqrt{1-\rho^{2}}),$$

with a covariance matrix:

$$\sum =\left[\begin{array}{cc} \sigma_{1}^{2} & \sigma_{12}\\ \sigma_{12} & \sigma_{2}^{2} \end{array}\right]=\left[\begin{array}{cc} \sigma_{1}^{2} & \rho \sigma_{1}\sigma_{2}\\ \rho \sigma_{1}\sigma_{2} & \sigma_{2}^{2} \end{array}\right].$$

**Figure 12:**
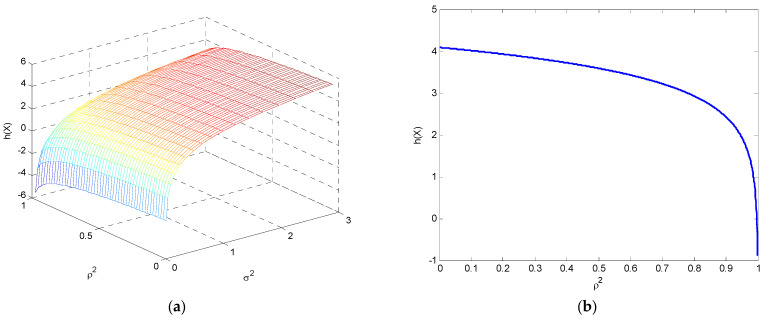
The variation in differential entropy for the bivariate Gaussian distribution (**a**) as a function of $\rho^{2}$ and $\sigma^{2}$, and (**b**) as a function of $\rho^{2}$ when $\sigma_{1}=\sigma_{2}=1$.

If $\sigma_{1}=\sigma_{2}=\sigma$, this becomes:

(37) $$h(X_{1},X_{2})=\log_{2}(2\pi e\sigma^{2}\sqrt{1-\rho^{2}}),$$

which is a function of $\rho^{2}$ concave downward and grows first very fast and then much more slowly for high $\rho^{2}$ values, as shown in Figure 12.

### 3.3. The Differential Entropy in the Transformed Frame

The differential entropy is invariant to a translation (change in the mean of the pdf):

h(X+a)=h(X),

and

$$h(bX)=h(X)+\log_{2}|b|.$$

For a random variable vector, the differential entropy in the transformed frame remains the same as the one in the original frame. It can be shown in general that:

(38) $$h(Y)=h(UX)=h(X)+\log_{2}|U|=h(X).$$

For the case of a multivariate Gaussian distribution, we have:

$$h(X)=\frac{1}{2}\log_{2}({\left(2\pi e\right)}^{n}|\sum |)=\frac{n}{2}\log_{2}(2\pi e)+\frac{1}{2}\log_{2}|\sum |=\frac{n}{2}\log_{2}(2\pi e)+\sum_{i=1}^{n}\frac{1}{2}\log_{2}\lambda_{i}$$

It is known that the determinant of the covariance matrix is equal to the product of its eigenvalues:

$$|\sum |=\prod_{i=1}^{n}\lambda_{i}.$$

For the case of a bivariate Gaussian distribution, n=2, we have:

(39) $$\begin{array}{l} f_{Y}(y)=\prod_{i=1}^{2}\frac{1}{\sqrt{2\pi }\sqrt{\lambda_{i}}}e^{-\frac{1}{2}\frac{y_{i}^{2}}{\lambda_{i}}}\\ =\frac{1}{\sqrt{2\pi }\sqrt{\lambda_{1}}}e^{-\frac{1}{2}\frac{y_{1}^{2}}{\lambda_{1}}}\cdot \frac{1}{\sqrt{2\pi }\sqrt{\lambda_{2}}}e^{-\frac{1}{2}\frac{y_{2}^{2}}{\lambda_{2}}}\\ =\frac{1}{2\pi \sqrt{\lambda_{1}\lambda_{2}}}e^{-\frac{1}{2}\left(\frac{y_{1}^{2}}{\lambda_{1}}+\frac{y_{2}^{2}}{\lambda_{2}}\right)} \end{array}.$$

It can be shown that the entropy in the transformed frame is given by:

$$h(Y)=\frac{2}{2}\log_{2}(2\pi e)+\sum_{i=1}^{2}\log_{2}(\lambda_{i})=\log_{2}(2\pi e)+\log_{2}(\lambda_{1}\cdot \lambda_{2}).$$

Detailed derivations are provided in Appendix D. As discussed, the determinant of the covariance matrix is equal to the product of its eigenvalues:

(40) $$\begin{array}{l} |\sum |=\lambda_{1}\cdot \lambda_{2}\\ =\left(\frac{1}{2}\left[\sigma_{1}^{2}+\sigma_{2}^{2}+\sqrt{{\left(\sigma_{1}^{2}-\sigma_{2}^{2}\right)}^{2}+4\sigma_{1}^{2}\sigma_{2}^{2}\rho^{2}}\right]\right)\left(\frac{1}{2}\left[\sigma_{1}^{2}+\sigma_{2}^{2}-\sqrt{{\left(\sigma_{1}^{2}-\sigma_{2}^{2}\right)}^{2}+4\sigma_{1}^{2}\sigma_{2}^{2}\rho^{2}}\right]\right)\\ =\sigma_{1}^{2}\sigma_{2}^{2}(1-\rho^{2}) \end{array},$$

and thus, the entropy can be presented as:

(41) $$h(Y_{1},Y_{2})=\frac{1}{2}\log_{2}{\left(2\pi e\right)}^{2}|\sum |=\frac{1}{2}\log_{2}{\left(2\pi e\right)}^{2}\sigma_{1}^{2}\sigma_{2}^{2}(1-\rho^{2}))=\log_{2}(2\pi e\sigma_{1}\sigma_{2}\sqrt{1-\rho^{2}}).$$

The result confirms the statement that the differential entropy remains unchanged in the transformed frame.

## 4. Relative Entropy (Kullback–Leibler Divergence)

In this section, various important issues regarding relative entropy (Kullback–Leibler divergence) are discussed. Despite the aforementioned flaws, there is a possibility of information theory in the continuous case. A key result is that the definitions for relative entropy and mutual information follow naturally from the discrete case and retain their usefulness.

The relative entropy is a type of statistical distance that provides a measure of probability distribution $f_{X}$, is different from a second reference probability distribution $g_{X}$, denoted as:

(42) $$D_{KL}(f\|g)=\int f_{X}(x)\log_{2}\frac{f_{X}(x)}{g_{X}(x)}dx.$$

A detailed derivation is provided in Appendix E. The relative entropy between two Gaussian distributions with different mean and variance is given by:

(43) $$D_{KL}(f\|g)=\frac{1}{2}\left[\ln\left(\frac{\sigma_{2}^{2}}{\sigma_{1}^{2}}\right)+\frac{\sigma_{1}^{2}}{\sigma_{2}^{2}}+{\left(\frac{\mu_{1}-\mu_{2}}{\sigma_{2}}\right)}^{2}-1\right]\cdot \log_{2}e.$$

**Figure 13:**
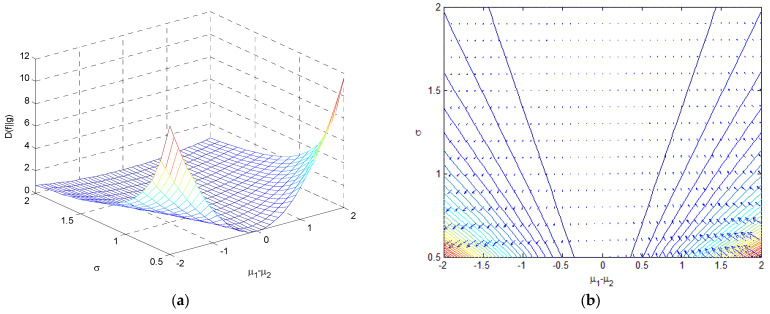
The variation in relative entropy as a function of σ and $\mu_{1}-\mu_{2}$ when $\sigma_{1}=\sigma_{2}=\sigma$ for (**a**) a three-dimensional surface; and (**b**) a contour with an entropy gradient.

**Figure 14:**
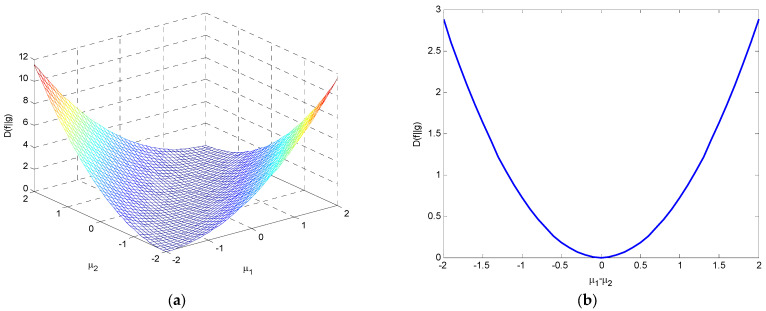
The variations in relative entropy with $\sigma_{1}=\sigma_{2}=1$ for (**a**) a three-dimensional surface as a function of $\mu_{1}$ and $\mu_{2}$; and (**b**) as a function of $\mu_{1}-\mu_{2}$.

**Figure 15:**
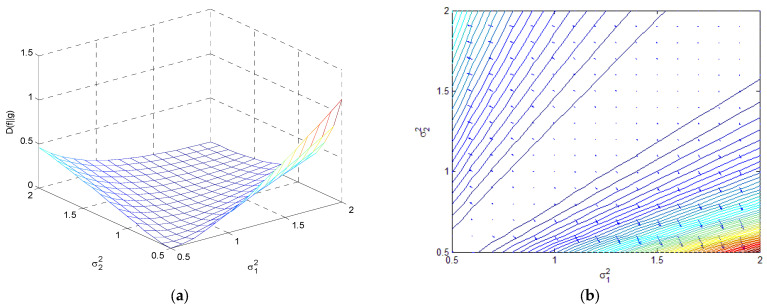
The variation in relative entropy as a function of $\sigma_{1}$ and $\sigma_{2}$ with $\mu_{1}=\mu_{2}$ for (**a**) at the three-dimensional surface; and (**b**) contour with an entropy gradient.

It is worth noting that the relative entropy measured in bits where $\log_{2}$ is used in the definition. However, if ln is used, then it would be measured in nats. The only difference in the expression is the $\log_{2}e$ factor. Several conditions are discussed with examples of the characteristics of relative entropy:

(1) If $\sigma_{1}=\sigma_{2}=\sigma$, $D_{KL}(f\|g)=\frac{1}{2}{\left(\frac{\mu_{1}-\mu_{2}}{\sigma }\right)}^{2}\log_{2}e$, which is 0 when $\mu_{1}=\mu_{2}$. Figure 13 shows the relative entropy as a function of σ and $\mu_{1}-\mu_{2}$ when $\sigma_{1}=\sigma_{2}=\sigma$, where a three-dimensional surface and a contour with an entropy gradient are provided.

(2) If $\sigma_{1}=\sigma_{2}=1$, $D_{KL}(f\|g)=\frac{1}{2}{\left(\mu_{1}-\mu_{2}\right)}^{2}\cdot \log_{2}e$, which is an even function with a minimum value of 0 when $\mu_{1}=\mu_{2}$. Figure 14 illustrates the variations of relative entropy as a function of $\mu_{1}$ and $\mu_{2}$ and as a function of $\mu_{1}-\mu_{2}$.

– If $\mu_{2}=0$, $D_{KL}(f\|g)=\frac{1}{2}\mu_{1}^{2}\log_{2}e$, it is a function of $\mu_{1}$ concave upward.
– If $\mu_{1}=0$, $D_{KL}(f\|g)=\frac{1}{2}\mu_{2}^{2}\log_{2}e$, it is a function of $\mu_{2}$ concave upward.

(3) If $\mu_{1}=\mu_{2}$, $D_{KL}(f\|g)=\frac{1}{2}\left[\ln\left(\frac{\sigma_{2}^{2}}{\sigma_{1}^{2}}\right)+\frac{\sigma_{1}^{2}}{\sigma_{2}^{2}}-1\right]\cdot \log_{2}e$. Figure 15 demonstrates relative entropy as a function of $\sigma_{1}$ and $\sigma_{2}$ when $\mu_{1}=\mu_{2}$, where a three-dimensional surface and the contour with an entropy gradient are plotted.

When $\sigma_{2}=1$, $D_{KL}(f\|g)=\frac{1}{2}\left[\ln\left(\frac{1}{\sigma_{1}^{2}}\right)+\sigma_{1}^{2}-1\right]\cdot \log_{2}e$.

When $\sigma_{1}=1$, $D_{KL}(f\|g)=\frac{1}{2}\left[\ln\left(\sigma_{2}^{2}\right)+\frac{1}{\sigma_{2}^{2}}-1\right]\cdot \log_{2}e$.

**Figure 16:**
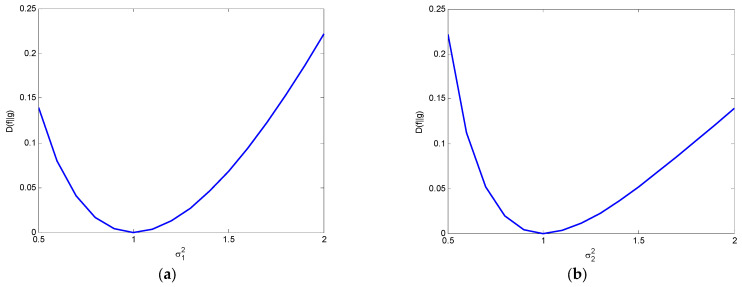
Variations of relative entropy as a function of (**a**) $\sigma_{1}$ when fixed $\sigma_{2}=1$ and (**b**) $\sigma_{2}$ when fixed $\sigma_{1}=1$, respectively ($\mu_{1}=\mu_{2}$).

Figure 16 illustrates the variations of relative entropy as a function of the variance when the other variance is unity under the condition $\mu_{1}=\mu_{2}$.

A sensitivity analysis of the relative entropy due to changes in variance and mean is carried out. The gradient of $D_{KL}(f\|g)$ given by:

$$\frac{\partial D_{KL}(\sigma_{1},\sigma_{2},\mu_{1},\mu_{2})}{\partial x}=\left[\begin{array}{cccc} \frac{\partial D_{KL}}{\partial \sigma_{1}} & \frac{\partial D_{KL}}{\partial \sigma_{2}} & \frac{\partial D_{KL}}{\partial \mu_{1}} & \frac{\partial D_{KL}}{\partial \mu_{2}} \end{array}\right],$$

can be calculated with the partial derivatives where the chain rule is involved. Based on the relationship $\frac{d}{dx}\ln x=\frac{1}{x}$, we have:

$$\frac{\partial }{\partial \sigma_{1}}\left[\ln\left(\frac{\sigma_{2}^{2}}{\sigma_{1}^{2}}\right)\right]=\frac{\sigma_{1}^{2}}{\sigma_{2}^{2}}\cdot (-2)\sigma_{2}^{2}\sigma_{1}^{-3}=-\frac{2}{\sigma_{1}},$$

and the following expressions are obtained:

(1) $\frac{\partial D_{KL}}{\partial \sigma_{1}}=\frac{\partial }{\partial \sigma_{1}}\left[\ln\left(\frac{\sigma_{2}^{2}}{\sigma_{1}^{2}}\right)+\frac{\sigma_{1}^{2}}{\sigma_{2}^{2}}\right]\cdot \frac{1}{2}\log_{2}e=\left(\frac{\sigma_{1}}{\sigma_{2}^{2}}-\frac{1}{\sigma_{1}}\right)\cdot \log_{2}e$,

(2) $\frac{\partial D_{KL}}{\partial \sigma_{2}}=\frac{\partial }{\partial \sigma_{2}}\left[\ln\left(\frac{\sigma_{2}^{2}}{\sigma_{1}^{2}}\right)+\frac{\sigma_{1}^{2}}{\sigma_{2}^{2}}+\frac{{\left(\mu_{1}-\mu_{2}\right)}^{2}}{\sigma_{2}^{2}}\right]\cdot \frac{1}{2}\log_{2}e=\left[\frac{1}{\sigma_{2}}-\frac{\sigma_{1}^{2}}{\sigma_{2}^{3}}-\frac{{\left(\mu_{1}-\mu_{2}\right)}^{2}}{\sigma_{2}^{3}}\right]\cdot \log_{2}e$,

(3) $\frac{\partial D_{KL}}{\partial \mu_{1}}=\frac{\partial }{\partial \mu_{1}}{\left(\frac{\mu_{1}-\mu_{2}}{\sigma_{2}}\right)}^{2}\cdot \frac{1}{2}\log_{2}e=\left(\frac{\mu_{1}-\mu_{2}}{\sigma_{2}^{2}}\right)\cdot \log_{2}e$,

(4) $\frac{\partial D_{KL}}{\partial \mu_{2}}=\frac{\partial }{\partial \mu_{2}}{\left(\frac{\mu_{1}-\mu_{2}}{\sigma_{2}}\right)}^{2}\cdot \frac{1}{2}\log_{2}e=\left(\frac{\mu_{2}-\mu_{1}}{\sigma_{2}^{2}}\right)\cdot \log_{2}e$.

The optimal condition for each of the above cases can be:

$\frac{\partial D_{KL}}{\partial \sigma_{1}}=\frac{\sigma_{1}}{\sigma_{2}^{2}}-\frac{1}{\sigma_{1}}=0$ when $\sigma_{1}^{2}=\sigma_{2}^{2}$,

$\frac{\partial D_{KL}}{\partial \sigma_{2}}=\frac{1}{\sigma_{2}}-\frac{\sigma_{1}^{2}}{\sigma_{2}^{3}}-\frac{{\left(\mu_{1}-\mu_{2}\right)}^{2}}{\sigma_{2}^{3}}=0$ when $\sigma_{2}^{2}=\sigma_{1}^{2}+{\left(\mu_{1}-\mu_{2}\right)}^{2}$,

$\left(\frac{\mu_{1}-\mu_{2}}{\sigma_{2}^{2}}\right)\cdot \log_{2}e=0$ when $\mu_{1}=\mu_{2}$,

$\left(\frac{\mu_{2}-\mu_{1}}{\sigma_{2}^{2}}\right)\cdot \log_{2}e=0$ when $\mu_{1}=\mu_{2}$.

## 5. Mutual Information

Mutual information is one of many quantities that measures one random variable and tells us about another. It is a dimensionless quantity with (generally) units of bits and can be thought of as the reduction in uncertainty about one random variable given knowledge of another. The mutual information I(X;Y) between two variables with joint pdf $f_{XY}(x,y)$ is given by:

(44) $$I(X;Y)=E\left[\log\frac{f_{XY}(x,y)}{f_{X}(x)f_{Y}(y)}\right]=\int \int f_{XY}(x,y)\log\frac{f_{XY}(x,y)}{f_{X}(x)f_{Y}(y)}dxdy.$$

The mutual information between the random variables *X* and *Y* has the following relationships:

(45) I(X;Y)=I(Y;X),

where

(46) I(X;Y)=h(X)−h(X|Y)≥0,

and

(47) I(Y;X)=h(Y)−h(Y|X)≥0,

implying that h(X)≥h(X|Y) and h(Y)≥h(Y|X). The mutual information of a random variable with itself is self-information, which is entropy. High mutual information indicates a large reduction in uncertainty; low mutual information indicates a small reduction; and zero mutual information between two random variables, I(X;Y)=0, meaning that the variables are independent. In such a case, h(X)=h(X|Y) and h(Y)=h(Y|X).

Let’s consider the mutual information between the correlated Gaussian variables *X* and *Y* given by:

(48) $$\begin{array}{l} I(X;Y)=h(X)+h(Y)-h(X,Y)\\ =\frac{1}{2}\log_{2}(2\pi e)\sigma_{x}^{2}+\frac{1}{2}\log_{2}(2\pi e)\sigma_{y}^{2}-\frac{1}{2}\log_{2}{\left(2\pi e\right)}^{2}\sigma_{x}^{2}\sigma_{y}^{2}(1-\rho^{2})\\ =-\frac{1}{2}\log_{2}(1-\rho^{2}) \end{array}.$$

**Figure 17:**
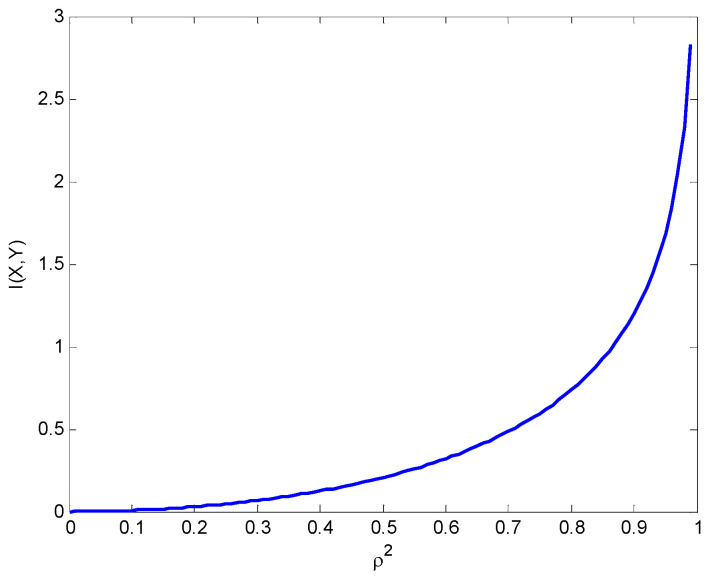
Mutual information versus $\rho^{2}$ between the correlated Gaussian variables.

Figure 17 presents the mutual information versus $\rho^{2}$, where it grows first much slower and then very fast for high values of $\rho^{2}$. If ρ=±1, the random variables *X* and *Y* are perfectly correlated, the mutual information is infinite. It can be seen that I(X;Y)=0 for ρ=0 and that I(X;Y)→∞ for ρ→±1.

**Figure 18:**
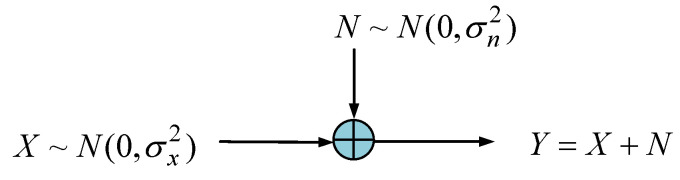
Schematic illustration of the additive white Gaussian noise (AWGN) channel.

On the other hand, considering the additive white Gaussian noise (AWGN) channel, shown in Figure 18, the mutual information is given by:

(49) $$I(X;Y)=h(Y)-h(Y|X)=\frac{1}{2}\log_{2}\left(\frac{2\pi e(\sigma_{x}^{2}+\sigma_{n}^{2})}{2\pi e\sigma_{n}^{2}}\right)=\frac{1}{2}\log_{2}\left(1+\frac{\sigma_{x}^{2}}{\sigma_{n}^{2}}\right),$$

where h(Y|X)=h(N)=h(X,Y)−h(X), and

$$h(Y)=\frac{1}{2}\log_{2}\left(2\pi e(\sigma_{x}^{2}+\sigma_{n}^{2})\right);\;h(Y|X)=h(N)=\frac{1}{2}\log_{2}(2\pi e)\sigma_{n}^{2}$$

**Figure 19:**
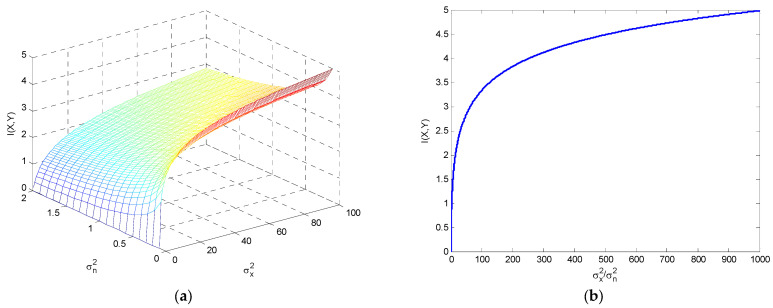
The mutual information with the additive white Gaussian noise (AWGN) channel for (**a**) the three-dimensional surface as a function of $\sigma_{x}^{2}$ and $\sigma_{n}^{2}$; and (**b**) in terms of the signal-to-noise ratio.

Mutual information for the additive white Gaussian noise (AWGN) channel is shown in Figure 19, including the three-dimensional surface as a function of $\sigma_{x}^{2}$ and $\sigma_{n}^{2}$, and also in terms of the signal-to-noise ratio $\mathrm{SNR}=\sigma_{x}^{2}/\sigma_{n}^{2}$. It can be seen that the mutual information grows first very fast and then much more slowly for high values of the signal-to-noise ratio.

## 6. Conclusions

This paper intends to serve the readers as a supplement note for the multivariate Gaussian distribution with its entropy, relative entropy, and mutual information. The illustrative examples are discussed to provide further insights into the geometric interpretation and visualization, enabling the readers to correctly interpret the theory for future design. The fundamental objective is to study the application of multivariate sets of data to a Gaussian distribution. This paper examines broad measurements of structure for Gaussian distributions, which show that they can be described in terms of the information theory between the given covariance matrix and correlated random variables (in terms of relative entropy). To develop the multivariate Gaussian distribution with entropy and mutual information, several significant methodologies are presented through the discussion supported by illustrations, both technically and statistically. The content obtained allows readers to better perceive concepts, comprehend techniques, and properly execute software programs for future study on the topic’s science and implementations. It also helps readers grasp the themes’ fundamental concepts. Involving the relative entropy and mutual information as well as the potential correlated covariance analysis based on differential equations, a wide range of information is addressed, from basic to application concerns. Moreover, the proposed techniques of multivariate Gaussian distribution and mutual information are intended to inspire new applications of information theory to a number of areas, including information coding, nonlinear signal detection, and clinical diagnostic testing, particularly when data from improved testing equipment becomes accessible.

## Data Availability

Not applicable.

## Appendix A. Derivation of the Differential Entropy for the Univariate Gaussian Distribution

$$\begin{array}{l} h(X)\\ =-E[\log_{2}f_{X}(x)]\\ =-\int f_{X}(x)\log_{2}f_{X}(x)dx\\ =-\int f_{X}(x)\log_{2}\left(\frac{1}{\sqrt{2\pi }\sigma }e^{-\frac{{\left(x-\mu \right)}^{2}}{2\sigma^{2}}}\right)dx\\ =-\int f_{X}(x)\left(\log_{2}{\left(2\pi \sigma^{2}\right)}^{-\frac{1}{2}}+\log_{2}e^{-\frac{{\left(x-\mu \right)}^{2}}{2\sigma^{2}}}\right)dx\\ =-\int f_{X}(x)\left[\left(-\frac{1}{2}\log_{2}(2\pi \sigma^{2})-\frac{{\left(x-\mu \right)}^{2}}{2\sigma^{2}}\log_{2}e\right)\right]dx\\ =\frac{1}{2}\log_{2}(2\pi \sigma^{2})\int_{-\infty }^{\infty }f_{X}(x)dx+\frac{\log_{2}e}{2\sigma^{2}}\int_{-\infty }^{\infty }{\left(x-\mu \right)}^{2}f_{X}(x)dx\\ =\frac{1}{2}\log_{2}(2\pi \sigma^{2})+\frac{\sigma^{2}}{2\sigma^{2}}\log_{2}e\\ =\frac{1}{2}\log_{2}(2\pi e\sigma^{2}) \end{array}$$

## Appendix B. Derivation of the Differential Entropy for the Multivariate Gaussian Distribution

$$\begin{array}{l} h(X)\\ =-E\left[\log_{2}f_{X}(x)\right]\\ =-E\left[\log_{2}\left(\frac{1}{\sqrt{{\left(2\pi \right)}^{n}|\sum |}}e^{-\frac{1}{2}{\left(x-\mu \right)}^{T}\sum^{-1}(x-\mu )}\right)\right]\\ =-E\left[-\frac{n}{2}\log_{2}(2\pi )-\frac{1}{2}\log_{2}|\sum |-\log_{2}e^{\frac{1}{2}{\left(x-\mu \right)}^{T}\sum^{-1}(x-\mu )}\right]\\ =-\int f_{X}(x)\left[\left(-\frac{1}{2}\log_{2}{\left(2\pi \right)}^{n}|\sum |)-\frac{\log_{2}e}{2}({\left(x-\mu \right)}^{T}\sum^{-1}(x-\mu ))\right)\right]dx\\ =\frac{1}{2}\log_{2}({\left(2\pi \right)}^{n}|\sum |)+\frac{n}{2}\log_{2}e\\ =\frac{n}{2}\log_{2}(2\pi )+\frac{1}{2}\log_{2}|\sum |+\log_{2}e^{\frac{1}{2}{\left(x-\mu \right)}^{T}\sum^{-1}(x-\mu )}\\ =\frac{n}{2}\log_{2}(2\pi )+\frac{1}{2}\log_{2}|\sum |+\log_{2}e^{\frac{n}{2}}\\ =\frac{n}{2}\log_{2}(2\pi )+\frac{1}{2}\log_{2}|\sum |+\frac{n}{2}\log_{2}e\\ =\frac{1}{2}\log_{2}\left({\left(2\pi e\right)}^{n}|\sum |\right) \end{array}$$

The calculation involves the evaluation of expectations of the Mahalanobis distance.

## Appendix C. Evaluation of Expectations of the Mahalanobis Distance $E[{\left(x-\mu \right)}^{T}\sum^{-1}(x-\mu )]=n$

$$\begin{array}{l} E[{\left(x-\mu \right)}^{T}\sum^{-1}(x-\mu )]\\ =E[tr({\left(x-\mu \right)}^{T}\sum^{-1}(x-\mu ))]\\ =E[tr(\sum^{-1}{\left(x-\mu \right)}^{T}(x-\mu ))]\\ =tr(\sum^{-1}E[{\left(x-\mu \right)}^{T}(x-\mu )])\\ =tr(\sum^{-1}\sum )\\ =tr(I_{n})\\ =n \end{array}$$

A special case for n=1

$$\begin{array}{l} E[{\left(x-\mu \right)}^{T}\sum^{-1}(x-\mu )]\\ =E\left[\frac{{\left(x-\mu \right)}^{2}}{\sigma^{2}}\right]\\ =\int f_{X}(x)\left(\frac{{\left(x-\mu \right)}^{2}}{\sigma^{2}}\right)dx\\ =\frac{1}{\sigma^{2}}\int {\left(x-\mu \right)}^{2}f_{X}(x)dx\\ =1 \end{array}$$

## Appendix D. Derivation of the Differential Entropy in the Transformed Frame

$$\begin{array}{l} h(Y)\\ =-E\left[\log_{2}\left(\prod_{i=1}^{n}\frac{1}{\sqrt{2\pi }\sqrt{\lambda_{i}}}e^{-\frac{1}{2}\frac{y_{i}^{2}}{\lambda_{i}}}\right)\right]\\ =-E\left[\sum_{i=1}^{n}\log_{2}\left(\frac{1}{\sqrt{2\pi }\sqrt{\lambda_{i}}}e^{-\frac{1}{2}\frac{y_{i}^{2}}{\lambda_{i}}}\right)\right]\\ =-\sum_{i=1}^{n}E\left[\log_{2}\left(\frac{1}{\sqrt{2\pi }\sqrt{\lambda_{i}}}e^{-\frac{1}{2}\frac{y_{i}^{2}}{\lambda_{i}}}\right)\right]\\ =-\sum_{i=1}^{n}E\left[\log_{2}(\frac{1}{\sqrt{2\pi }\sqrt{\lambda_{i}}})+\log_{2}e^{-\frac{1}{2}\frac{y_{i}^{2}}{\lambda_{i}}}\right]\\ =-\sum_{i=1}^{n}\left[\int f_{Y}(y_{i})\left(\log_{2}(\frac{1}{\sqrt{2\pi }\sqrt{\lambda_{i}}})+\log_{2}e^{-\frac{1}{2}\frac{y_{i}^{2}}{\lambda_{i}}}\right)dy_{i}\right]\\ =-\sum_{i=1}^{n}\left[\int f_{Y}(y_{i})\left(-\frac{1}{2}\log_{2}(2\pi \lambda_{i})-\frac{1}{2}\frac{y_{i}^{2}}{\lambda_{i}}\log_{2}e\right)dy_{i}\right]\\ =\sum_{i=1}^{n}\left[\frac{1}{2}\log_{2}(2\pi \lambda_{i})+\frac{1}{2}\log_{2}e\right]\\ =\sum_{i=1}^{n}\left[\frac{1}{2}\log_{2}(2\pi )+\frac{1}{2}\log_{2}(\lambda_{i})+\frac{1}{2}\log_{2}e\right]\\ =\frac{n}{2}\log_{2}(2\pi e)+\frac{1}{2}\sum_{i=1}^{n}\log_{2}(\lambda_{i}) \end{array}$$

The eigenvalues $\lambda_{i}$ are the diagonal elements of the covariance matrix, namely variances, in the transformed frame. When ρ=0, the eigenvectors are equal to $\lambda_{i}=\sigma_{i}^{2}$.

## Appendix E. Derivation of the Kullback–Leibler Divergence between Two Normal Distributions

$$\begin{array}{l} D_{KL}(f\|g)\\ =\int f_{X}(x)\log_{2}\frac{f_{X}(x)}{g_{X}(x)}dx\\ =\int f_{X}(x)\log_{2}\frac{\frac{1}{\sqrt{2\pi }\sigma_{1}}e^{-\frac{1}{2}{\left(\frac{x-\mu_{1}}{\sigma_{1}}\right)}^{2}}}{\frac{1}{\sqrt{2\pi }\sigma_{2}}e^{-\frac{1}{2}{\left(\frac{x-\mu_{2}}{\sigma_{2}}\right)}^{2}}}dx\\ =\int f_{X}(x)\log_{2}\left(\frac{\sigma_{2}}{\sigma_{1}}\right)dx+\int f_{X}(x)\log_{2}\left[\exp\left(-\frac{1}{2}{\left(\frac{x-\mu_{1}}{\sigma_{1}}\right)}^{2}+\frac{1}{2}{\left(\frac{x-\mu_{2}}{\sigma_{2}}\right)}^{2}\right)\right]dx\\ =\log_{2}\left(\frac{\sigma_{2}}{\sigma_{1}}\right)-\frac{\log_{2}e}{2\sigma_{1}^{2}}\int f_{X}(x){\left(x-\mu_{1}\right)}^{2}dx+\frac{\log_{2}e}{2\sigma_{2}^{2}}\int f_{X}(x){\left(x-\mu_{2}\right)}^{2}dx\\ =\log_{2}\left(\frac{\sigma_{2}}{\sigma_{1}}\right)-\frac{\log_{2}e}{2}+\frac{\log_{2}e}{2\sigma_{2}^{2}}\int f_{X}(x){\left(x-\mu_{1}+\mu_{1}-\mu_{2}\right)}^{2}dx\\ =\log_{2}\left(\frac{\sigma_{2}}{\sigma_{1}}\right)-\frac{\log_{2}e}{2}+\frac{\log_{2}e}{2\sigma_{2}^{2}}\int f_{X}(x)\left(x-\mu_{1}{)}^{2}+{\left(\mu_{1}-\mu_{2}\right)}^{2}+2(x-\mu_{1})(\mu_{1}-\mu_{2})\right)dx\\ =\frac{1}{2}\log_{2}\left(\frac{\sigma_{2}^{2}}{\sigma_{1}^{2}}\right)-\frac{\log_{2}e}{2}+\frac{\log_{2}e}{2\sigma_{2}^{2}}\left[\sigma_{1}^{2}+{\left(\mu_{1}-\mu_{2}\right)}^{2}\right]\\ =\frac{1}{2}\left[\ln\left(\frac{\sigma_{2}^{2}}{\sigma_{1}^{2}}\right)+\frac{\sigma_{1}^{2}}{\sigma_{2}^{2}}+{\left(\frac{\mu_{1}-\mu_{2}}{\sigma_{2}}\right)}^{2}-1\right]\cdot \log_{2}e \end{array}$$

where equality $\log_{2}(\cdot )=\log_{2}e\cdot \ln(\cdot )$ was used.
